# Effects of citral on serum antioxidant status and liver genes expressions of *paraoxonase 1* and *nitric oxide synthase* in a rat model of streptozotocin-induced diabetes mellitus

**DOI:** 10.22099/ijvr.2021.38416.5585

**Published:** 2021

**Authors:** M. Khosravi Bakhtiari, H. Sharifiyazdi, S. Nazifi, M. Ghaemi, M. Hadadipour Zarandi

**Affiliations:** 1 Resident of Clinical Pathology, Department of Clinical Sciences, School of Veterinary Medicine, Shiraz University, Shiraz, Iran;; 2 Department of Clinical Sciences, School of Veterinary Medicine, Shiraz University, Shiraz, Iran;; 3 Department of Pathobiology, School of Veterinary Medicine, Shiraz University, Shiraz, Iran

**Keywords:** Citral, Diabetes, Gene expression, Nitric oxide synthase, Paraoxonase 1

## Abstract

**Background:**

Citral (C_10_H_16_O) is the main ingredient of *Cymbopogon citratus* (lemongrass oil) and can reduce the side effects of oxidative stress. Diabetes caused by insulin deficiency induces oxidative stress in the liver.

**Aims:**

This study aimed to investigate the ameliorative effects of citral on selected oxidative parameters and the gene expression of *paraoxonase 1* (*PON1*) and *endothelial nitric oxide synthase* (*eNOS*) in a rat model of streptozotocin (STZ)-induced diabetes mellitus.

**Methods:**

Forty rats were divided into four groups at random: control (C), control citral (CC), and two STZ-induced diabetic groups (diabetic (D) and citral diabetic (CD)). After diabetes confirmation (day 7), gavage treatment with citral (300 mg/kg body weight (BW)) was started in the CD and CC groups and continued for two weeks.

**Results:**

On day 21 of the study, following treatment with citral for 14 days, the serum levels of total antioxidant capacity (TAC), and PON1 in the CD group were significantly increased compared to those in the D group (P<0.05). While treatment with citral caused a significant decrease in the Malondialdehyde (MDA), and eNOS in the CD group compared to those of the D group (P<0.001). The expression rate of liver *PON1* gene was considerably upregulated in the CD group compared to that in the D group (P<0.001); while the opposite was observed for *eNOS* gene expression. However, there was no significant difference between the CC and C groups in terms of all examined parameters (P>0.05).

**Conclusion:**

This study showed positive effects of citral on serum antioxidant status and liver gene expression of *PON1* and *eNOS* in diabetic rats.

## Introduction

 Citral (C_10_H_16_O) is the main ingredient of *Cymbopogon citratus* (lemongrass oil) (Tajidin et al., 2012 ▶) and has been used in perfumery, cosmetic, and pharmaceutical industries to control both bacterial and fungal infections (Giteru et al., 2015 ▶). In addition, citral has been proposed as a hepatoprotective and renoprotective agent in mice due to its anti-inflammatory and antioxidant properties (Yang et al., 2013 ▶; Lai et al., 2016 ▶). A few studies have shown that citral reduces the detrimental effects of systemic diseases by decreasing oxidative stress and apoptosis levels (Yang et al., 2013 ▶). In a preliminary *in vitro* study, citral was identified as an inducer of total and pi-class-specific activity of glutathione S-transferase (GST) in RL34 rat liver epithelial cells (Nakamura et al., 2003 ▶). Accordingly, it has been suggested that citral may have a key role in the antioxidant defense processes and detoxifying reactions (Li et al., 2018 ▶). A previous study showed that the citral can also reduce oxidant activity as well as macrophage, and NF-κB activation (Yang et al., 2013 ▶).

 Diabetic complications and the subsequent oxidative stress are a growing concern worldwide. Diabetes caused by insulin deficiency induces oxidative stress in several ways (Kaneto et al., 2007 ▶). Recent data suggest that chronic hyperglycemia contributes to oxidative events by enhancing excessive free radical production, particularly reactive oxygen species (ROS) (Hossein-Nia et al., 2018 ▶). Accordingly, prevention or reduction of oxidative stress could represent an appropriate therapeutic approach for the complications of oxidative stress in diabetes (Förstermann et al., 2017 ▶). Evidence shows that the paraoxonase (PON) enzyme family plays an important role in diminishing oxidative stress and lipid peroxidation. In particular, paraoxonase 1 (PON1) and PON3 reside on high-density lipoprotein (HDL) and can prevent the atherosclerosis development (Förstermann et al., 2017 ▶).

 Evidence of decreased PON1 activity is well recognized in diabetes (Flekac et al., 2008 ▶). Paraoxonase 1 is predominantly expressed in hepatocytes and is associated with HDL; its activity is responsible for the anti-atherogenic and cardioprotective characteristics of HDL (Förstermann et al., 2017 ▶). Paraoxonase 1 catalyzes the hydrolysis of aromatic carboxylic acid esters, organophosphate esters, and carbamates (Flekac et al., 2008 ▶).

 Besides, there has been increasing evidences that nitric oxide synthase (NOS) has a pivotal role in diabetic complications. The endothelial nitric oxide synthase (eNOS) enzyme is constitutively and mainly expressed in endothelial cells under physiological conditions and its activity is regulated by the shear stress of the flowing blood and by mediators, such as acetylcholine and bradykinin. This enzyme produces endothelial NO that induces relaxation of vascular smooth muscle and prevents platelet activation. Endothelial NO also inhibits leukocyte adhesion to the vascular wall and leukocyte migration into the vascular endothelium. Furthermore, eNOS can inhibit the oxidation of low-density lipoproteins (LDL), as well as preventing the proliferation of the vascular smooth muscle cells (Förstermann et al., 2017 ▶).

 Due to the altered expressions of *PON1* and *eNOS* in patients with diabetes mellitus (DM) and the association between the serum changes in these enzymes and oxidative stress indices, citral is assumed to affect the gene expressions of these enzymes in liver at the molecular level. Accordingly, we aimed to evaluate the effects of citral on the activities of PON1 and eNOS and on some oxidative stress parameters in serum of rats with streptozotocin (STZ)-induced diabetes mellitus. The gene expressions of liver *PON1* and *eNOS* were also evaluated uing quantitative real-time polymerase chain reaction (qPCR).

## Materials and Methods

**Animals**

 Forty male Wistar rats (200 ± 12 g) were provided from the Center of Laboratory Animals of the Faculty of Veterinary Medicine of Shahid Bahonar University, Kerman, Iran. Rats were housed in a temperature-controlled room at 23 ± 1°C with 12-hour light/dark cycles and had free access to rat chow (Pars, Iran) and water *ad libitum*. The animals experienced seven days of acclimatization before the beginning of the study.

**Animal ethics**

 The protection of animals used in this experiment were approved by the State Committee on Animal Ethics, Shiraz University, Shiraz, Iran (IACUC No.: 4687/63). The approvals of the European Council Directive (2010/63/EU) of Sept., 12, 2010, were also followed.

**Citral**

 Citral (mixture of cis and trans isomers) was provided from Merk KGaA (Darmstadt, Germany) with a catalog number of 802489.

**Experimental design**

 Forty rats were randomly divided into four groups: control (C), control citral (CC), diabetic (D), and citral diabetic (CD). There were two control (C and CC), and two diabetic (D and CD) groups. Each of the two diabetic groups was treated with two injections of STZ (45 mg/kg) (Sigma, Germany), which was dissolved in 0.05 M citrate buffer (pH = 4.5) and administered with a 24-hour interval (Bhattacharya, 1995 ▶). To confirm diabetes induction, the blood glucose level was measured by a glucometer (EasyGluco, South Korea) five days after the second injection. At this period, the hyperglycemia above 300 mg/dL confirmed the induction of diabetes mellitus. At the following day, the two groups of CD and CC gavaged continuously with citral (300 mg/kg body weight (BW)) for 2 weeks (Uchida et al., 2017a ▶, b ▶). Citral was administered with corn oil, as a vehicle; in the other two groups, C and D, the corn oil was gavaged, separately.

**Sampling**

 Blood samples were taken from all animals via heart puncture under light diethyl ether anesthesia on days 0 (before the injection of STZ), 7, and 21. On day 21, animals were euthanized with ether and the liver were immediately sampled. Sera were separated and stored at -20°C.

**Serum biochemical assays**

 Serum glucose was measured using a commercial kit based on the glucose oxidase method (Pars Azmoon Co., Tehran, Iran) with the auto-analyzer Alpha Classic AT++ (Sanjesh Co., Isfahan, Iran). Insulin, PON1, and eNOS were measured in the serum based on a quantitative sandwich enzyme immunoassay method using commercial species-specific enzyme-linked immunosorbent assay (ELISA) kits for rats (Shanghai Crystal Day Biotech, Shanghai, China). A sensitivity of 0.75 μ IU/ml was reported for the insulin assay as per the commercial ELISA kit. The inter- and intra-assay precisions of the insulin kit were coefficient of variation (CV) < 10% and CV < 8%, respectively. The sensitivity of the PON1 ELISA kit was 0.53 ng/ml. The inter- and intra-assay precisions of the PON1 ELISA kit were CV < 10% and CV < 8%, respectively. The sensitivity of the eNOS ELISA kit was 0.26 ng/ml and its inter- and intra-assay precisions were CV < 10% and CV < 8%, respectively. Malondialdehyde (MDA) and total antioxidant capacity (TAC) were also quantified using a commercial kit (ZellBio GmbH, Germany) as described by the manufacturer.

**Isolation of total RNA and synthesis of cDNA**

 The animals were euthanized with diethyl ether on day 21. Total RNA was extracted from 100 mg of liver tissue using RiboEx^TM^ total RNA buffer (GeneAll, South Korea) according to the manufacturer’s procedure. Possible DNA contamination was removed after treating RNA (1 μg) with DNase I (2 U/μL) at 37°C for 1 h (GeneAll, South Korea). The purity and quantity of the extracted RNA were determined using a NanoDrop spectrophotometer at 60 nm wavelength. To determine the purity of RNA, its optical density (OD) ratio at 260/280 nm was determined and the samples with OD ratio >1.8 were used for reverse transcription. cDNA synthesis conducted using the PrimeScript^TM^ RT kit with random hexamer primers and 1 μg of RNA according to the kit protocol of the instruction (Takara, Japan). During cDNA synthesis, No-RT controls were also used in each round to check the contamination of the genomic DNA in real-time PCR. cDNAs were stored at -20°C until used for real-time PCR.

**Table 1:** The primer sequence and product size used in real-time PCR for the genes expressions

Primer	Oligo	Sequence	Amplicon size (bp)	Reference
*PON1*	Forward	5´-TGGCATTGGCATTTCCCTTGA-3´	125	Designed in this study
*PON1*	Reverse	5´-TGTCAAAGCTGAGGACCTTCAAT-3´		Designed in this study
*NOS*	Forward	5´-CTGCGGTGATGTCACTATGG-3´	140	Rashed *et al*. (2016)
*NOS*	Reverse	5´-AAATGTCCTCGTGGTAGCGT-3´		Rashed *et al*. (2016)
*GAPDH*	Forward	5´-CAGTGCCAGCCTCGTCTCAT-3´	197	Zhang *et al*. (2015)
*GAPDH*	Reverse	5´-GTGCCGTTGAACTTGCCGTG-3´		Perrault *et al*. (2020)

PCR: Polymerase chain reaction

**Real-time PCR analysis**

 The relative real-time qPCR analysis of target genes (*PON1* and *eNOS*) using *GAPDH* as an internal reference gene was done in a LightCycler 96 (Roche, Germany). The specific sets of primers used for this experiment are presented in Table 1.

 Final master mix volume for the analysis of gene expression was 20 μL, containing 4 μL qPCR TM Green Master kit for EVA Green I (Solis BioDyne, Estonia), 2 μL cDNA (~100 ng), 0.4 μL of forward and reverse primers (200 nM), and 13.2 μL nuclease-free distilled water. The PCR cycling conditions were 95°C for 15 min, followed by 45 cycles at 95°C for 15 s, and 60°C for 30 s. The PCR reactions were performed in triplicate. All runs contained one negative-template control, consisting of PCR-grade water instead of cDNA and No-RT control. Melting curve analysis was also performed in a final step to confirm the speciﬁcity of each PCR product. Relative quantification was performed to validate the assay to ensure that the primers used for the internal reference and target genes had similar amplification efficiencies.

 The comparative 2^-ΔΔCt^ method was used to determine the relative gene expression values of the experimental samples compared to the control samples using REST2009 software (Qiagen, USA). The experimental practices and data analysis related to quantitative reverse transcription PCR (RT-qPCR) were provided based on the minimum information for publication of real-time qPCR experiments (MIQE) guidelines.

**Statistical analysis**

 Analyses were carried out using statistical software SPSS version 25. Descriptive statistic was provided as means ± standard error (SE). The means for all parameters in groups at various time points were compared by applying one way ANOVA method, followed by the Tukey post-hoc test. The transformation of data was performed in the case of non-homogeneous variance and high variability. For most parameters, the variance became homogenous after logarithmic transformation. In all analyses, s**ignificance was declared at P**≤**0.05.**

## Results

**Glucose and insulin levels in serum**

**Fig. 1:**
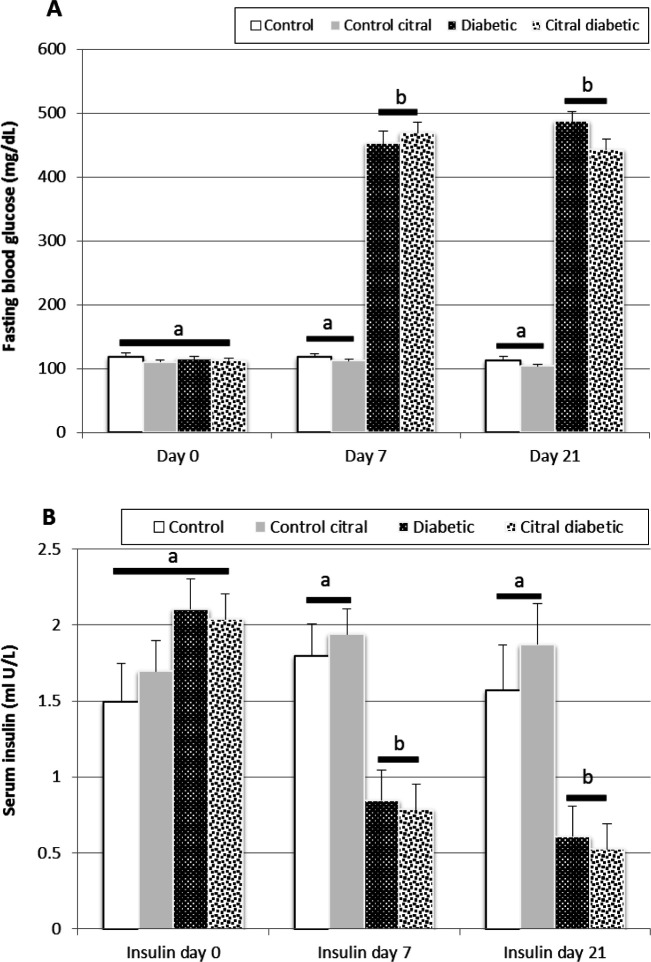
A comparison of mean fasting blood glucose (**A**) and insulin (**B**) levels in the control (C), control citral (CC), diabetic (D), and citral diabetic (CD) groups on different days. Data are expressed as means±SE. Different letters (a, b) demonstrate significant differences (P<0.05) between groups on each day

 The results of glucose and insulin assays in this study were obtained on days 0, 7, and 21. Comparisons of biochemical parameters between groups showed that on day 0, there was no significant difference between the four groups (Figs. 1A and B). On day 7, a significant increase was observed in serum glucose levels after the STZ injection and development of diabetes in the D and CD groups compared to the C and CC groups (Fig. 1A).

 Lower levels of insulin were observed in the diabetic groups (D and CD) after diabetes induction compared to the control groups (C and CC) (P=0.001, Fig. 1B). These results showed that STZ treatment induced diabetes and resulted in lower insulin and higher glucose (>300 mg/dL) levels compared to the control animals. The results on day 21 showed that the treatment of the CD group for 14 days did not improve hyperglycemia and hypoinsulinemia (Figs. 1A-B).

**Serum biochemical**

**Fig. 2:**
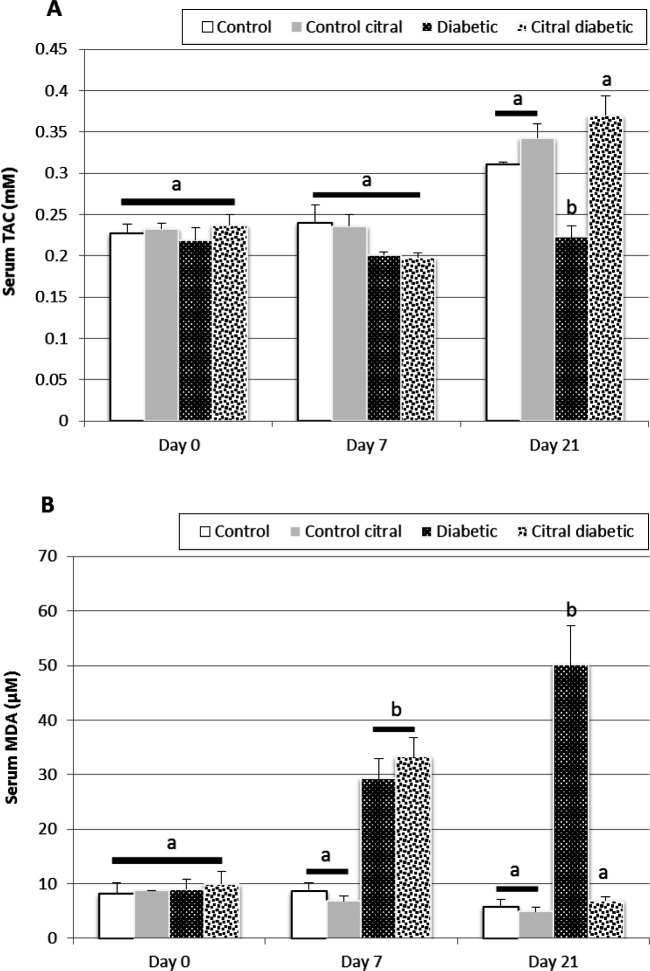
A comparison of serum total antioxidant capacity (TAC) (**A**) and serum malondialdehyde (MDA) (**B**) in the control (C), control citral (CC), diabetic (D), and citral diabetic (CD) groups on different days. Data are expressed as means±SE. Different letters (a, b) demonstrate significant differences (P<0.05) between groups on each day

 The changes in oxidative markers (TAC and MDA) on days 0, 7, and 21 at various time points are shown in Figs. 2A and B. On day 0, no significant difference was observed between the four groups in the above-mentioned oxidative markers. However, on day 7, a significantly higher level of serum MDA was observed in the diabetic groups (D and CD) compared to the control groups (C and CC) (P<0.05). Conversely, the TAC level in diabetic rats was lower than that of control animals on day 7. At the end of the treatment with citral (day 21), the serum level of MDA significantly decreased in the CD group compared to that of the D group (P<0.05). However, on day 21, the serum level of TAC significantly increased in the CD group compared to that of the D group (P<0.05).

**Fig. 3:**
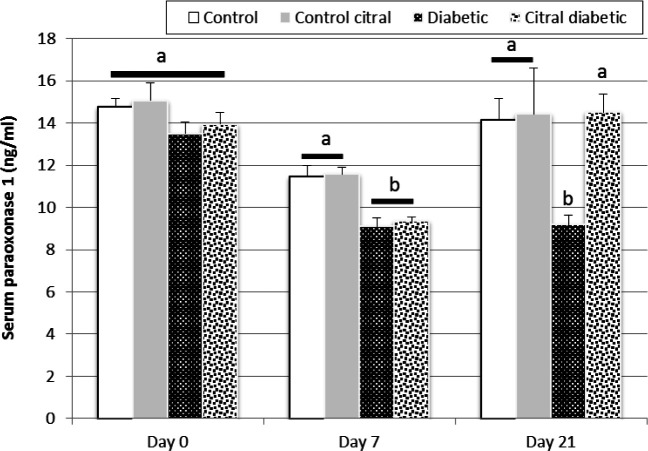
A comparison of the serum levels of paraoxonase 1 (PON1) in the control (C), control citral (CC), diabetic (D), and citral diabetic (CD) groups on different days. Data are expressed as means±SE. Different letters (a, b) demonstrate significant differences (P<0.05) between groups on each day

 There was no significant difference between the four groups in PON1 serum activity on day 0. On day 7 of the study, the serum level of PON1 significantly decreased in diabetic groups after the induction of diabetes compared to that of the control groups (P=0.003). However, the CD group treatment for 14 days caused a significant increase in the serum levels of PON1 on day 21 compared to the D group which did not receive any treatment (P=0.007, Fig. 3).

**Real-time PCR**

**Fig. 4:**
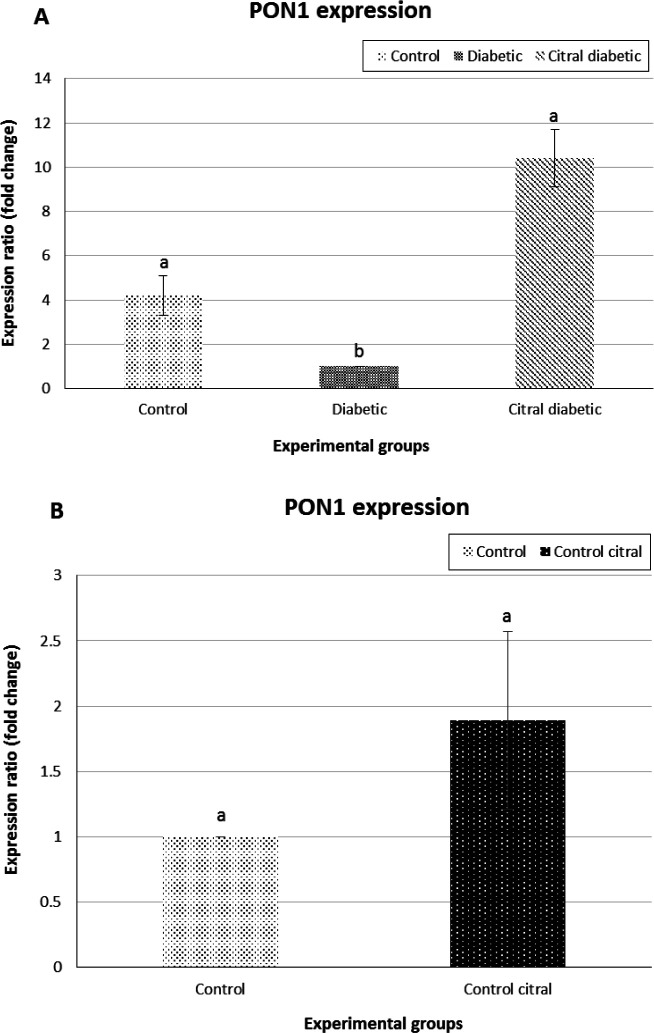
Real-time PCR results of *paraoxonase 1* (*PON1*). (**A**) A comparison of *PON1* gene expression level of the liver tissue among control (C), diabetic (D), and citral diabetic (CD) groups (diabetic group considered as calibrator). Data are expressed as means±SE. Different letters (a, b) demonstrate significant differences among groups (P<0.05), and (**B**) A comparison of *PON1* gene expression of the liver tissue between C and control citral (CC) groups (control group considered as calibrator)

 The real-time PCR results showed that the expression level of liver *PON1* was significantly downregulated in D group on day 21 compared to that of the C group. In addition, on day 21, the expression level of liver *PON1* was significantly upregulated in CD group compared to D group (P=0.0001, Fig. 4A). Moreover, similar comparison was made between the C and CC groups concerning the above-mentioned gene. The findings revealed no significant difference between these two groups in the mRNA expression levels of liver *PON1* (P=0.99, Fig. 4B).

**Fig. 5:**
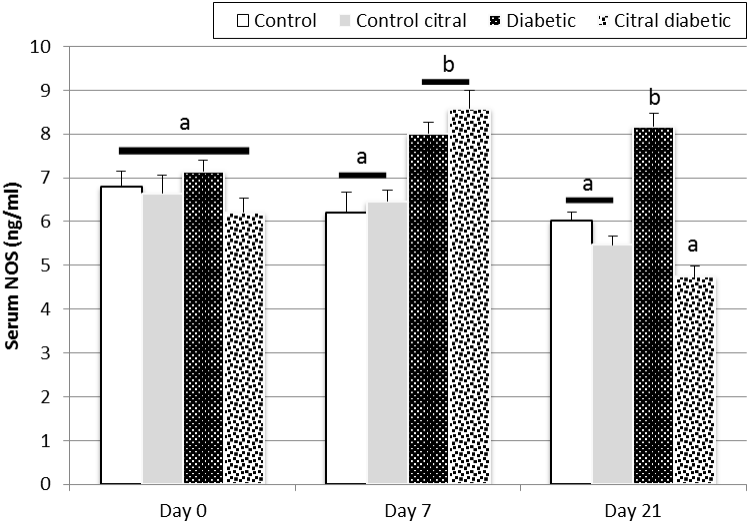
The effects of citral on the serum nitric oxide synthase (NOS) activities in control (C), control citral (CC), diabetic (D), and citral diabetic (CD) rats on different days. Data are expressed as means±SE. Different letters (a, b) demonstrate significant differences (P<0.05) between groups on each day

 There was no significant difference between the four groups in the serum NOS activity on day 0. The serum NOS activity in diabetic rats (D and CD) significantly increased on day 7 compared to that in the control groups (C and CC). However, the treatment of CD group for 14 days caused a significant decrease in the serum NOS activity on day 21 of the experiment compared to that of the D group (P<0.05, Fig. 5).

**Fig. 6:**
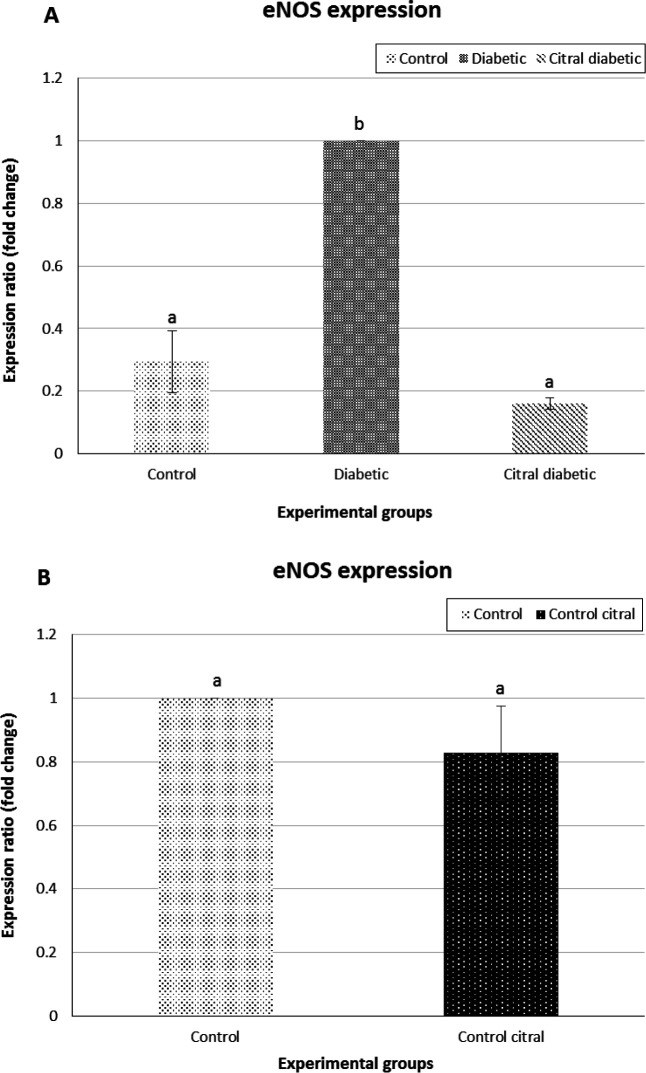
Real-time PCR results of *endothelial nitric oxide synthase* (e*NOS*). (**A**) A comparison of e*NOS* expression gene of the liver tissue among control (C), diabetic (D), and citral diabetic (CD) groups (diabetic group considered as calibrator). Data are expressed as means±SE. Different letters (a, b) demonstrate significant differences among groups (P<0.05), and (**B**) A comparison of e*NOS* gene expression of the liver tissue between C and control citral (CC) groups (control group considered as calibrator)

 However, on day 21, the expression level of liver *eNOS* was significantly downregulated in the CD group compared to that of the D group (P<0.05, Fig. 6A). 

While the results showed no significant difference between the control groups (C and CC) on day 21 in terms of mRNA expression levels of *eNOS* (P>0.05, Fig. 6B).

## Discussion

 This study examined the ameliorative effects of treatment with citral, on the attenuation of oxidative stress in a rat model of STZ-induced diabetes mellitus.

 Several studies have examined the antioxidative and anti-inflammatory effects of citral, but the mechanisms behind these effects have remained unclear. Uchida et al. (2017) ▶ showed that citral could reduce hepatic toxicity induced by acetaminophen (Uchida et al., 2017a ▶).

 To investigate the effects of citral, we examined some oxidative markers, including MDA, TAC, PON, and NOS, and the genes expressions of two important enzymes, i.e., *PON1* and *eNOS*, in liver tissue. The findings showed a significant increase in the serum glucose, MDA, and NOS levels on day 7 in the diabetic groups (D and CD) compared to the control groups (C and CC). Conversely, there was a significant decrease in the serum insulin, PON, and TAC levels on day 7 in the diabetic groups (D and CD) compared to the control groups (C and CC).

 Several studies showed that oxidant/antioxidant balance is disturbed due to the oxidative stress and this balance increased production of free radical products under hyperglycemia in diabetic cases (Scott and King, 2004 ▶; Yamagishi and Imaizumi, 2005 ▶; Förstermann et al., 2017 ▶).

 Likewise, our findings showed that the serum levels of PON1 and TAC decreased after the induction of diabetes in rats. Similarly, Karabina et al. (2005) reported that PON1 activity decreased in diabetes mellitus (Flekac et al., 2008 ▶); consequently, an increased risk of oxidative stress occurred in the diabetic patients (James et al., 2000 ▶). These changes may lead to further complications. First, the rise in lipid peroxidation increases oxidative stress and causes vascular wall injury (Gross et al., 2003 ▶). Second, the proteins glycation, including enzymes, may decrease the activity of proteins in diabetes (Maritim et al., 2003; Flekac et al., 2008 ▶). The increased expression of *PON1*, as an antioxidant enzyme, results in reduced lipoprotein oxidation in patients with diabetes (James et al., 2000 ▶). Decreased activity of the PON1 enzyme may lead to a reduction in the HDL ability to inhibit LDL oxidation and to increase atherosclerosis, which is associated with diabetes (Mackness et al., 2002 ▶). Reduced PON1 activity in diabetic patients was found to be associated with increased risk of oxidative stress, allowing the conclusion that PON1 activity is a predictor of oxidative stress in diabetic patients (Kota et al., 2013 ▶). According to previous studies, PON1 dysregulates the expression or secretion patterns in the liver of diabetic rats (Kao et al., 1998 ▶). Although *PON1* overexpression reduces atherosclerosis, any disruption in the expression of *PON1* in oxidative stress exacerbates atherogenesis (De Vries et al., 2000 ▶). The results of the present study revealed that *PON1* gene expression in liver tissue was upregulated in the citral-treated diabetic rats and its enzyme activity increased in the serum following consumption of citral. These findings suggested an ameliorative effect of citral on the serum antioxidant status and the liver genes expressions of connected enzymes.

 Paraoxonase 1 activity was reported to decrease in patients with diabetes (Siewert et al., 2015 ▶). Flekac et al. (2008) ▶ also observed a negative correlation between serum PON1 activity in both types of diabetes mellitus and the levels of glycated hemoglobin (Flekac et al., 2008 ▶). Decreased PON1 activity is associated with increased lipid-peroxide formation, and accelerated oxidative stress (Flekac et al., 2008 ▶). The results of the MDA assay in the present study support the above-mentioned findings. It seems that the gavage treatment of the CD group decreased the MDA production induced by high glucose through the up-regulation of *PON1* in the liver. In another study, a diet supplemented with lemongrass oil (containing citral) increased the activity of oxidative stress enzymes and reduced the MDA level in heat-stressed growing rabbits (Al-Sagheer et al., 2017 ▶). Several studies have reported an increase in oxidative stress and a higher susceptibility to lipid peroxidation of LDL in diabetic patients compared with healthy individuals. Oxidative stress has been recognized as one of the principal causes of atherogenic modifications in LDL and, consequently, of atherosclerotic disease (Zaki et al., 2014 ▶). Paraoxonase 1 can inhibit lipid peroxides accumulation in LDL, due to its ability to reduce hydroperoxides, and can attenuate the biological effects of mildly oxidized LDL. Both HDL and LDL isolated from *PON1*-knockout were equally susceptible to oxidation (Flekac et al., 2008 ▶). In particular, studies in *PON*-knockout mice have revealed that the absence of *PON1* is associated with higher susceptibility of lipoproteins to oxidation and more extensive atherosclerotic plaques. Decreased PON1 can increase the risk of LDL oxidation, and, due to the preponderant atherogenic role of oxidized LDL, LDL oxidation can increase the risk of vascular disease (Sharma et al., 2015 ▶). Our results showed that the PON1 activity in serum increased after the citral gavage corresponding to the up-regulation of *PON1* in the liver. Consequently, this study represents an alternative therapeutic approach for the reduction of the mentioned-diabetic complications using herbal compound by increasing PON1 activity. Nevertheless, the results revealed that citral did not affect glucose and insulin levels in serum of the diabetic groups (D and DC). Similar studies also showed the hepatoprotective effects of citral against hepatocyte injury caused by acetaminophen during an experimental model (Uchida et al., 2017a ▶, b ▶). The citral hepatoprotective effect can be attributed to either the reduction of oxidative stress following its treatment or the anti-inflammatory properties of citral (Yang et al., 2013 ▶; Lai et al., 2016 ▶; Uchida et al., 2017b ▶). Also, in an *in vitro* study, was shown that citral reduce ROS and prevent LDL oxidation during hyperglycemia and induction of hydrogen peroxide (Campos et al., 2014 ▶).

 In this study, the gene expression and activity of NOS increased after diabetes induction in rats. However, we found a decrease in the serum NOS activity and tissue expression of this enzyme in the CD group. Several mechanisms, such as hyperglycemia, have been implicated in reduced endothelium-derived NO availability and, at the same time, increased formation of oxidative stress in diabetes (De Vries et al., 2000 ▶). Reactive oxygen species modulate vascular tone by scavenging NO and producing peroxynitrite (ONOO^-^). ONOO^-^, as one of the endogenous ROS, oxidizes proteins, causes breakage in DNA, and leads to the depletion of intracellular antioxidants, such as cysteine and glutathione. Reactive oxygen species also contribute to the oxidative modification of LDL (Ülker et al., 2003 ▶).

 Unexpectedly, the eNOS expression contributes to increase atherogenesis under pathological conditions. These paradoxical findings are explained by later results that eNOS overexpression leads to eNOS uncoupling owing to a relative deficiency of eNOS cofactor BH4. The separation of eNOS is a critical factor in the production of atherosclerosis. This pathological situation not only reduces endothelium-derived NO availability but also exacerbates existing oxidative stress (Li et al., 2014 ▶). Accordingly, the eNOS upregulation in diabetes and downregulation after citral administration in this study can be justified. NO is also recognized as an inflammatory marker and the prevention of its production can be considered as a suitable therapeutic approach in inflammatory conditions (Uchida et al., 2017b ▶). In one study, the activities of iNOS and eNOS were significantly decreased in the hippocampus and cerebral cortex in diabetic rats after treatment with astaxanthin (Xu et al., 2015 ▶). In another study, bee venom was used as an anti-inflammatory agent to heal diabetic wounds in mice, and the results showed that it could reduce the expression of *NOS* gene in the wounded tissue of diabetic mice (Badr et al., 2016 ▶).

 Likewise, some studies have shown that citral can act as a cell protector against oxidant agents in the liver (Li et al., 2018 ▶). Some studies have revealed that citral can inhibit the release of important inflammatory cytokines, such as IL-1β, IL-6, TNF-a, and NO. It is suggested that citral plays its anti-inflammatory role through the inhibition of NO production (Uchida et al., 2017b ▶).

 This study revealed that treatment with citral could have noticeable antioxidant activity in diabetes. The gavage of citral prevented oxidative stress alterations caused by diabetes, suppressed the increase in MDA (an oxidative stress marker), and attenuated the decrease of PON1 and TAC in diabetic rats serums.

 Several studies have indicated the hepatoprotective effects of *C. citratus* oil on hepatotoxicity in the rats or mice experimental models (Rahim et al., 2014 ▶; Saenthaweesuk et al., 2017 ▶; Uchida et al., 2017b ▶). The findings of our study suggested that the improvement in oxidative stress following pretreatment with lemongrass essential oil can be attributed to citral as the major constituent of this plant essential oil.

 Our findings revealed that the expression level of liver *PON1* increased following gavage treatment with citral in the STZ-induced diabetic rats; this change was in parallel with the increased serum activity of this enzyme despite high blood glucose levels. Also, the data obtained from other oxidative stress markers in the serum allow us to suggest citral as a substance that may be beneficial in reducing the complications of oxidative stress in diabetes. Furthermore, citral decreased *eNOS* gene expression in live tissue. This effect of citral could be associated with either its role in reducing oxidative stress or its inhibitory impact on inflammatory events.

## References

[B1] Al-Sagheer AA, Daader AH, Gabr HA, Abd El-Moniem EA (2017). Palliative effects of extra virgin olive oil, gallic acid, and lemongrass oil dietary supplementation on growth performance, digestibility, carcass traits, and antioxidant status of heat-stressed growing New Zealand White rabbits. Environ. Sci. Pollut. Res.

[B2] Badr G, Hozzein WN, Badr BM, Al Ghamdi A, Saad Eldien HM, Garraud O (2016). Bee venom accelerates wound healing in diabetic mice by suppressing activating transcription factor 3 (ATF 3) and inducible nitric oxide synthase (iNOS) mediated oxidative stress and recruiting bone marrow derived endothelial progenitor cells. J. Cell. Physiol.

[B3] Bhattacharya SK (1995). Shilajit attenuates streptozotocin induced diabetes mellitus and decrease in pancreatic islet superoxide dismutase activity in rats. Phytother. Res.

[B4] Campos J, Schmeda-Hirschmann G, Leiva E, Guzman L, Orrego R, Fernandez P, González M, Radojkovic C, Zuñiga F, Lamperti L (2014). Lemon grass (Cymbopogon citratus (DC) Stapf) polyphenols protect human umbilical vein endothelial cell (HUVECs) from oxidative damage induced by high glucose, hydrogen peroxide and oxidised low-density lipoprotein. Food Chem.

[B5] De Vries A, Verbeuren T, Van de Voorde J, Lameire N, Vanhoutte P (2000). Endothelial dysfunction in diabetes. Brit. J. Pharmacol.

[B6] Flekac M, Skrha J, Zidkova K, Lacinova Z, Hilgertova J (2008). Paraoxonase 1 gene polymorohisms and enzyme activities in diabetes mellitus. Physiol. Res.

[B7] Förstermann U, Xia N, Li H (2017). Roles of vascular oxidative stress and nitric oxide in the pathogenesis of atherosclerosis. Circ. Res.

[B8] Giteru SG, Coorey R, Bertolatti D, Watkin E, Johnson S, Fang Z (2015). Physicochemical and antimicrobial properties of citral and quercetin incorporated kafirin-based bioactive films. Food Chem.

[B9] Gross D, Fogg L, Webster-Stratton C, Garvey C, Julion W, Grady J (2003). Parent training of toddlers in day care in low-income urban communities. J. Consult. Clin. Psych.

[B10] Hossein-Nia B, Khorram S, Rezazadeh H, Safaiyan A, Ghiasi R, Tarighat-Esfanjani A (2018). The effects of natural clinoptilolite and nano-sized clinoptilolite supplementation on lipid profile, food intakes and body weight in rats with streptozotocin-induced diabetes. Can. J. Diabetes.

[B11] James RW, Leviev I, Ruiz J, Passa P, Froguel P, Garin MC (2000). Promoter polymorphism T (-107) C of the paraoxonase PON1 gene is a risk factor for coronary heart disease in type 2 diabetic patients. Diabetes..

[B12] Kaneto H, Katakami N, Kawamori D, Miyatsuka T, Sakamoto K, Matsuoka T, Matsuhisa M, Yamasaki Y (2007). Involvement of oxidative stress in the pathogenesis of diabetes. Antiox. Redox. Sign.

[B13] Kao Y, Donaghue K, Chan A, Knight J, Silink M (1998). A variant of paraoxonase (PON1) gene is associated with diabetic retinopathy in IDDM. J. Clin. Endocr. Metab.

[B14] Kota SK, Meher LK, Kota SK, Jammula S, Krishna S, Modi KD (2013). Implications of serum paraoxonase activity in obesity, diabetes mellitus, and dyslipidemia. Indian J. Endocr. Metab.

[B15] Lai YS, Lee WC, Lin YE, Ho CT, Lu KH, Lin SH, Panyod S, Chu YL, Sheen LY (2016). Ginger essential oil ameliorates hepatic injury and lipid accumulation in high fat diet-induced nonalcoholic fatty liver disease. J. Agri. Food Chem.

[B16] Li H, Horke S, Förstermann U (2014). Vascular oxidative stress, nitric oxide and atherosclerosis. Atherosclerosis.

[B17] Li CC, Yu HF, Chang CH, Liu YT, Yao HT (2018). Effects of lemongrass oil and citral on hepatic drug-metabolizing enzymes, oxidative stress, and acetaminophen toxicity in rats. J. Food Drug Anal.

[B18] Mackness B, Durrington P, Boulton A, Hine D, Mackness M (2002). Serum paraoxonase activity in patients with type 1 diabetes compared to healthy controls. Eur. J. Clin Invest.

[B19] Magenta A, Greco S, Capogrossi MC, Gaetano C, Martelli F (2014). Nitric oxide, oxidative stress, and p66Shc interplay in diabetic endothelial dysfunction. Bio. Med. Res. Int.

[B20] Maritim A, Dene B, Sanders R, Watkins III J (2003). Effects of pycnogenol treatment on oxidative stress in streptozotocin-induced diabetic rats. J. Biochem. Mol. Toxcic.

[B21] Nakamura Y, Miyamoto M, Murakami A, Ohigashi H, Osawa T, Uchida K (2003). A phase II detoxification enzyme inducer from lemongrass: identification of citral and involvement of electrophilic reaction in the enzyme induction. Biochem. Bioph. Res. Co.

[B22] Perrault DP, Lee GK, Bouz A, Sung C, Yu R, Pourmoussa AJ, Park SY, Kim GH, Jiao W, Patel KM (2020). Ischemia and reperfusion injury in superficial inferior epigastric artery-based vascularized lymph node flaps. PLoS One.

[B23] Rahim SM, Taha EM, Al-Janabi MS, Al-Douri BI, Simon KD, Mazlan AG (2014). Hepatoprotective effect of Cymbopogon citratus aqueous extract against hydrogen peroxide-induced liver injury in male rats. Afr. J. Tradit. Complem.

[B24] Rashed L, Gharib DM, Hussein RE, Tork O, Abusree A (2016). Combined effect of bone marrow derived mesenchymal stem cells and nitric oxide inducer oninjured gastric mucosa in a rat model. Tissue Cell.

[B25] Saenthaweesuk S, Munkong N, Parklak W, Thaeomor A, Chaisakul J, Somparn N (2017). Hepato-protective and antioxidant effects of Cymbopogon citratus Stapf (Lemon grass) extract in paracetamolinduced hepatotoxicity in rats. Trop. J. Pharm. Res.

[B26] Scott JA, King GL (2004). Oxidative stress and antioxidant treatment in diabetes. Ann. NY. Acad. Sci.

[B27] Sharma A, Sellers S, Stefanovic N, Leung C, Tan SM, Huet O, Granville DJ, Cooper ME, de Haan JB, Bernatchez P (2015). Direct endothelial nitric oxide synthase activation provides atheroprotection in diabetes-accelerated atherosclerosis. Diabetes.

[B28] Siewert S, Gonzalez II, Lucero RO, Ojeda MS (2015). Association of cholesteryl ester transfer protein genotypes with paraoxonase 1 activity, lipid profile and oxidative stress in type 2 diabetes mellitus: A study in SanLuis, Argent. J. Diabetes Invest.

[B29] Tajidin N, Ahmad S, Rosenani A, Azimah H, Munirah M (2012). Chemical composition and citral content in lemongrass (Cymbopogon citratus) essential oil at three maturity stages. Afr. J. Biotech.

[B30] Uchida NS, Silva Filho SE, Aguiar RP, Wiirzler LAM, Cardia GFE, Cavalcante HAO, Silva Comar FMDS, Becker TCA, Silva EL, Bersani Amado CA (2017a). Protective effect of Cymbopogon citratus essential oil in experimental model of acetaminophen-induced liver injury. Am. J. Chinese Med.

[B31] Uchida NS, Silva Filho SE, Cardia GFE, Cremer E, Silva Comar FMDS, Silva EL, Bersani Amado CA, Cuman RKN (2017b). Hepatoprotective effect of citral on acetaminophen-induced liver toxicity in mice. Evid. Based. Comp. Alt. Med.

[B32] Ülker S, McKeown PP, Bayraktutan U (2003). Vitamins reverse endothelial dysfunction through regulation of eNOS and NAD (P) H oxidase activities. Hypertension.

[B33] Xu L, Zhu J, Yin W, Ding X (2015). Astaxanthin improves cognitive deficits from oxidative stress, nitric oxide synthase and inflammation through upregulation of PI3K/Akt in diabetes rat. Int. J. Clin. Exp. Patho.

[B34] Yamagishi Si, Imaizumi T (2005). Diabetic vascular complications: pathophysiology, biochemical basis and potential therapeutic strategy. Curr. Pharm.

[B35] Yang SM, Hua KF, Lin YC, Chen A, Chang JM, Chao LK, Ho CL, Ka SM (2013). Citral is renoprotective for focal segmental glomerulosclerosis by inhibiting oxidative stress and apoptosis and activating Nrf2 pathway in mice. PLoS One.

[B36] Zaki ME, El Bassyouni H, Kamal S, El Gammal M, Youness E (2014). Association of serum paraoxonase enzyme activity and oxidative stress markers with dyslipidemia in obese adolescents. Indian J. Endocrinol. Metab.

